# Besonderheiten und Probleme der Psychopharmakologie im Kindes- und Jugendalter

**DOI:** 10.1007/s00103-023-03718-z

**Published:** 2023-06-05

**Authors:** Manfred Gerlach, Tobias Renner, Marcel Romanos

**Affiliations:** 1 Klinik und für Kinder- und Jugendpsychiatrie, Psychosomatik und Psychotherapie Zentrum für Psychische Gesundheit, Universitätsklinik Würzburg, Margarete-Höppel-Platz 1, 97080 Würzburg, Deutschland; 2 Klinik für Psychiatrie, Psychosomatik und Psychotherapie im Kindes- und Jugendalter, Universitätsklinikum Tübingen, Tübingen, Deutschland; 3 Arbeitsgruppe „Kinder- und jugendliche Psychopharmakologie der Arbeitsgemeinschaft für Neuropsychopharmakologie und Pharmakopsychiatrie“ (AGNP) e. V., Homburg/Saar, Deutschland

**Keywords:** Arzneimittelzulassung, Off-Label-Anwendung, Pharmakovigilanz, Unerwünschte Arzneimittelwirkungen, Wirksamkeit, Drug approval, Off-label use, Pharmacovigilance, Adverse drug reactions, Efficacy

## Abstract

Die medikamentöse Behandlung von psychischen Erkrankungen im Kindes- und Jugendalter stellt eine besondere klinische und rechtliche Herausforderung dar. Gründe hierfür sind u. a. die häufig notwendigen Off-Label-Anwendungen und bestehende Wissenslücken in Bezug auf die Langzeitwirkungen der verwendeten Neuro‑/Psychopharmaka. In diesem Beitrag werden besondere Voraussetzungen der Therapie mit Neuro‑/Psychopharmaka, wie z. B. die Notwendigkeit der altersgerechten Einbeziehung der Kinder und Jugendlichen in den Entscheidungs- und Aufklärungsprozess sowie der Evaluation der Medikation, die Berücksichtigung biologischer alters- und reifungsabhängiger Faktoren und die besonderen Maßnahmen bei einer Off-Label-Anwendung besprochen. Weiterhin werden allgemeine Probleme bei der Entwicklung und Anwendung von Neuro‑/Psychopharmaka, wie etwa Schwierigkeiten in Bezug auf Wirknachweis, erstattungs- und haftungsrechtliche Fragen bei der Off-Label-Anwendung und die Problematik der Durchführung klinischer Studien im Kindes- und Jugendalter, diskutiert.

## Abstract (en)

The drug treatment of mental illness in childhood and adolescence poses a particular clinical and legal challenge. Reasons for this include the often necessary off-label use and existing knowledge gaps regarding the long-term effects of the neuro-/psychotropic drugs used. In this article, the prerequisites for therapy with neuro/psychotropic drugs, such as the need for age-appropriate inclusion of children and adolescents in the decision-making and education process, as well as the evaluation of medication, the consideration of biological age- and maturation-related factors, and the special measures for off-label use, are discussed. We further discuss general problems in the development and use of neuro-/psychotropic drugs, such as the difficulties in relation to proof of effectiveness, reimbursement and liability issues of off-label administration, and the problems of conducting clinical trials with children and adolescents.

## Einleitung

Die *Psychopharmakologie* ist ein Teilgebiet der Neuropharmakologie, das sich u. a. mit Fragestellungen der Anwendung von Medikamenten beschäftigt, die vorwiegend eine Wirkung auf das Gehirn ausüben und psychische Prozesse wie Erleben und Verhalten direkt beeinflussen (Neuro‑/Psychopharmaka; [1]).

Neuro‑/Psychopharmaka werden bei spezifischen Symptomen vieler kinder- und jugendpsychiatrischer Erkrankungen eingesetzt, wie bei der Aufmerksamkeits-Defizit/Hyperaktivitätsstörung (ADHS), Tic-Störungen, depressiven Störungen, schizophrenen Erkrankungen, Zwangs- und Angsterkrankungen, aber auch bei einzelnen transdiagnostischen Symptomen wie erhöhter Impulsivität, z. B. bei Störungen des Sozialverhaltens, Autismus-Spektrum-Störungen oder bei emotional instabilen Persönlichkeitsstörungen. Ihre Anwendung ist jedoch in Deutschland immer in ein therapeutisches Gesamtkonzept eingebettet, welches auch Psychoedukation, Psychotherapie und soziotherapeutische Maßnahmen vorsieht [2, 3].

In diesem Beitrag werden besondere Voraussetzungen der Therapie mit Neuro‑/Psychopharmaka im Kindes- und Jugendalter besprochen sowie Probleme bei der Anwendung und Entwicklung dargestellt. Ausführlichere Informationen zu diesen Themen finden sich zusätzlich in den Kapiteln „Entwicklungspsychiatrie“ [2] und „Rechtliche und ethische Fragen im klinischen Alltag“ [3] des Lehrbuchs *Neuro‑/Psychopharmaka im Kindes- und Jugendalter*: *Grundlagen und Therapie* und im Editorial „Medikamentöse Behandlung in der Kinder- und Jugendpsychiatrie in Deutschland zwischen ethischen, sozial- und haftungsrechtlichen Konflikten“ [4].

## Besonderheiten der Psychopharmakologie bei Kindern und Jugendlichen

Die medikamentöse Behandlung von psychischen Störungen im Kindes- und Jugendalter stellt eine besondere Herausforderung dar. Bis auf einzelne Ausnahmen ist die Datenlage zur Wirksamkeit und Sicherheit der meisten Neuro‑/Psychopharmaka in dieser Altersgruppe limitiert und nur eine sehr beschränkte Anzahl der verfügbaren Wirkstoffe ist für Personen unter 18 Jahren und nur für einzelne Indikationen zugelassen [4]. Kinder und Jugendliche weisen entwicklungsabhängige Stoffwechselbedingungen auf, deren Auswirkungen auf die Pharmakokinetik und Pharmakodynamik von Neuro‑/Psychopharmaka nur unzureichend untersucht sind [2]. Die mangelhafte Datenlage bezieht sich jedoch nicht nur auf die Off-Label-Behandlung, zumal auch Arzneimittel mit alten Zulassungen oftmals unzureichend hinsichtlich der altersabhängigen Besonderheiten von Wirkung und unerwünschten Arzneimittelwirkungen (UAWs) untersucht sind. Damit ergeben sich nicht nur für die Off-Label-Anwendung von Neuro‑/Psychopharmaka und insbesondere für die Polypharmazie unklare Risiken für Kinder und Jugendliche mit psychischen Erkrankungen.

### Aufklärung und Einverständnis von Sorgeberechtigten und Patientinnen und Patienten

Voraussetzung für eine Therapie mit Neuro‑/Psychopharmaka sind eine der Indikationsstellung zugrunde liegende, leitliniengetreue Diagnostik sowie die Aufklärung und Einwilligung der Betroffenen und ihrer Sorgeberechtigten [3]. Nur im Notfall (bei akuter Selbst- oder Fremdgefährdung) kann sich die Notwendigkeit ergeben, allein aufgrund des psychopathologischen Befundes eine Entscheidung zur symptomatischen medikamentösen Behandlung auch ohne Rechtsgrundlage zu treffen. Diese akuten Krisensituationen im Sinne des § 34 Strafgesetzbuch – StGB („Gefahr im Verzug“) unterliegen im Nachgang grundsätzlich einer richterlichen Überprüfung.

Unabdingbar sind die Einbeziehung des Kindes und insbesondere der oder des Jugendlichen in den Entscheidungs- und Aufklärungsprozess sowie die Evaluation einer geplanten Medikation, aber auch die Erörterung über ihre Fortsetzung. Mit zunehmender Reife haben Jugendliche das Recht, über die Pharmakotherapie mitzuentscheiden oder eigenständig zu entscheiden [3]. Vor Vollendung des 15. Lebensjahres existiert in der klinischen Praxis keine definierte Altersgrenze, ab der Kinder und Jugendliche für sich selber entscheiden dürfen. Vielmehr muss deshalb in Einzelfallentscheidungen die Reife eines Kindes individuell geprüft werden [3]. Ab dem vollendeten 15. Lebensjahr haben Jugendliche entsprechend § 36 SGB I das Recht, Leistungen zu beantragen und zu erhalten – und damit auch Leistungen wie die Verordnung von Arzneimitteln. Im Einzelfall können sich daraus in der Praxis komplexe Konstellationen ergeben, wenn Minderjährige eine medikamentöse Therapie wünschen bzw. ablehnen und dies dem Willen der Sorgeberechtigten widerspricht. Auch bei Uneinigkeit der Sorgeberechtigten ergeben sich aufwendige und bisweilen konflikthafte Entscheidungsprozesse.

Es gelten weiterhin die Regelungen des Patientenrechtegesetzes, wie sie etwa in den §§ 630 d‑e Bürgerliches Gesetzbuch (BGB) niedergelegt sind [3]. In die kinder- und jugendpsychiatrische Diagnostik und Behandlung müssen sowohl die Sorgeberechtigten als auch das Kind oder der bzw. die Jugendliche einwilligen, unabhängig davon, ob dies im (teil-)stationären oder ambulanten Setting erfolgt.

Sorgeberechtigte können eine Behandlung ihres Kindes, das die erforderliche Reife bereits erlangt hat, gegen seinen erklärten Willen nur bei akuter Eigen- oder Fremdgefährdung durchsetzen, indem sie eine familiengerichtliche Entscheidung gemäß §1631 b BGB herbeiführen. Unterbringungen und Zwangsbehandlungen nach den länderspezifischen Psychisch-Kranken-Gesetzen sind im Kindes- und Jugendalter grundsätzlich zu vermeiden und sollten nur speziellen Krisensituationen vorbehalten bleiben.

Die Interessen der Sorgeberechtigten können dabei dem geäußerten Kindeswillen und vor allem dem Kindeswohl widersprechen, z. B. wenn die Eltern nicht in der Lage sind, für das Kind Sorge zu tragen (z. B. infolge einer eigenen psychiatrischen Erkrankung) oder wenn Eltern dem Kindeswohl zuwiderhandeln (z. B. wenn eine wichtige medizinische Intervention dem Kind vorenthalten wird).

Um eine adäquate Einwilligung und Zustimmung geben zu können, ist die Information durch die behandelnde Person entscheidend. Leitfragen, die dabei helfen können, Eltern, Sorgeberechtigten und Minderjährigen eine umfassende und rechtlich hinreichende Aufklärung zu geben, sind ausführlich im Kapitel „Rechtliche und ethische Fragen im klinischen Alltag“ [3] des Lehrbuchs *Neuro‑/Psychopharmaka im Kindes- und Jugendalter*: *Grundlagen und Therapie* beschrieben.

### Berücksichtigung alters- und reifungsabhängiger Faktoren bei der medikamentösen Therapie im Kindes- und Jugendalter

Bei der medikamentösen Behandlung von Kindern und Jugendlichen sind insbesondere die Einflüsse der alters- und geschlechtsabhängigen körperlichen Entwicklung auf die Wirkung und Verträglichkeit von Neuro‑/Psychopharmaka zu berücksichtigen [2]. Die Therapie steht somit immer in Bezug zum Alter sowie dem biologischen und psychosozialen Entwicklungsstand der Patientin bzw. des Patienten. Dies kann bedeuten, dass physiologische Reifungsvorgänge die Wirksamkeit der Medikamente verändern. So kann grundsätzlich nicht davon ausgegangen werden, dass die Wirkung eines Arzneimittels bei einem Kleinkind die gleiche ist wie bei einem 17-jährigen Jugendlichen. Bei längerfristigen Therapien können außerdem Reifungsvorgänge oder psychosoziale Faktoren für die beobachteten klinischen Veränderungen wichtiger sein als die Wirkung eines Neuro‑/Psychopharmakons.

Abhängig von entwicklungsbedingten Besonderheiten von Kindern und Jugendlichen ist die Therapie mit Neuro‑/Psychopharmaka mit UAWs verbunden, die sich in Häufigkeit, Schweregrad und Art von denen bei Erwachsenen unterscheiden können. Kinder und Jugendliche weisen z. B. unter der Therapie mit bestimmten Antipsychotika im Vergleich zu Erwachsenen ein höheres Risiko für endokrin-metabolische Störungen (z. B. Gewichtszunahme, Prolaktinerhöhung, Entwicklung eines Diabetes mellitus) sowie für neurologische Komplikationen (z. B. Schläfrigkeit oder extrapyramidal-motorische Symptome) auf [5–8]. Bei der Einnahme von bestimmten Antidepressiva wird im Kindes- und Jugendalter ein erhöhtes Risiko für Suizidgedanken und suizidales Verhalten diskutiert [9]. Auch der subjektiv wahrgenommene Schweregrad von UAWs und ihre Auswirkungen auf das tägliche Leben bei jungen Patientinnen und Patienten können sich erheblich von denen bei Erwachsenen unterscheiden.

In vielen Fällen müssen Dosierungen bei Kindern und Jugendlichen deshalb vorsichtiger durchgeführt werden, da bei manchen Neuro‑/Psychopharmaka schon bei niedrigeren Dosierungen UAWs auftreten. Umgekehrt benötigen Kinder bei anderen Wirkstoffen eine höhere Arzneimitteldosis pro kg Körpergewicht als Erwachsene. Zu empfehlen ist deshalb die Durchführung eines therapeutischen Drug-Monitorings (TDM) zur Optimierung des Therapieeffektes und als proaktives Instrument zur Förderung der Pharmakovigilanz.

### Häufige Off-Label-Anwendung

Der behandelnde Arzt/die behandelnde Ärztin ist aus ethischen Gründen verpflichtet, den Patientinnen und Patienten eine wirksame und bestmögliche Behandlung zukommen zu lassen. Die Vorenthaltung einer indizierten Behandlung bei Kindern und Jugendlichen stellt eine Diskriminierung Minderjähriger dar [4]. Da die Mehrzahl der in Deutschland zur Behandlung von psychisch kranken Kindern und Jugendlichen angewandten Neuro‑/Psychopharmaka nicht für die betroffenen Altersstufen oder die jeweilig behandelte Indikation zugelassen sind, erfolgt diese als Off-Label-Anwendung unter bestimmten Voraussetzungen im Rahmen eines individuellen Heilversuches. Eine Off-Label-Anwendung bedeutet, dass ein Arzneimittel abweichend von den im Rahmen der Zulassung benannten Anwendungsgebieten eingesetzt wird, also einen „zulassungsüberschreitenden Gebrauch“. Dies kann neben den Indikationen auch die altersbezogene Anwendung, Dosierung und Art oder Dauer der Anwendung betreffen [10].

Die Off-Label-Verordnung eines Neuro‑/Psychopharmakons kann auch aus ethischen Gründen erforderlich sein, wenn das Nutzen-Risiko-Verhältnis einer zugelassenen Medikation im Vergleich zu einer nicht zugelassenen Medikation verschlechtert ist. Beispielsweise ist Haloperidol in Deutschland zwar für die Behandlung von Tic-Störungen bei stark beeinträchtigten Kindern und Jugendlichen im Alter von 10–17 Jahren nach Versagen anderer Therapiearten zugelassen, jedoch wird es aufgrund seiner extrapyramidal-motorischen UAWs nicht zur Behandlung empfohlen [11].

Generell gilt beim Off-Label-Einsatz eines Neuro‑/Psychopharmakons wie für alle anderen therapeutischen Interventionen auch, dass die Aussicht auf Wirkung für eine spezifische Symptomatik erwartbar ist, es also zumindest eine hinreichende Evidenz für den Einsatz gibt [2–4]. Der behandelnde Arzt/die behandelnde Ärztin muss im Einzelfall eine sorgfältige Risiko-Nutzen-Abwägung hinsichtlich der Vorteile und Risiken des Einsatzes eines Medikamentes treffen und diese Abwägung auch gemeinsam mit den Sorgeberechtigten und dem Patienten bzw. der Patientin erörtern. Auch die regelmäßige Einnahme, die Überwachung von UAWs durch Bezugspersonen sowie die Wahrnehmung von Kontrollterminen beim Verordnenden müssen gegeben sein. Bei Jugendlichen ist zudem zu beachten, dass keine gleichzeitige Einnahme von mit der Medikation interagierenden Substanzen stattfindet. Um die Wirksamkeit im Verlauf beurteilen zu können, ist es wichtig, Zielsymptome für die Pharmakotherapie eindeutig zu definieren und auch durch den Einsatz von standardisierten Instrumenten, beispielsweise Fragebögen/Checklisten, zu operationalisieren. TDM trägt dazu bei, Risiken von UAWs zu vermindern und den individuellen Therapieerfolg zu dokumentieren und zu optimieren.

## Probleme bei der Anwendung und Entwicklung von Neuro‑/Psychopharmaka

### Probleme des Nachweises der Wirksamkeit

Inwiefern ein Neuro‑/Psychopharmakon die erwartete Wirkung aufweist, wird im klinischen Alltag durch Exploration der Bezugspersonen und des Kindes oder auch systematischer durch Patient-reported Outcomes (PROMs) und zum Teil durch psychometrische Verfahren erfasst. Bei der Erhebung von UAWs sind neben Exploration und Fragebögen auch objektive Parameter (z. B. Blutwerte) von Bedeutung. Grundsätzlich muss die Beurteilung über längere Zeiträume hinweg erfolgen, zumal in der Psychopharmakotherapie im Kindes- und Jugendalter eine Reihe von Besonderheiten besteht, die die Wirkungsbeurteilung und Erfassung von UAWs verkomplizieren. Manche Betroffene weisen störungsbedingt eine schwankende Krankheits- und Symptomeinsicht auf, mit schwankender Fähigkeit und Bereitschaft zum Erkennen und Mitteilen von Symptomen. Auch die Beurteilung von Symptomen durch Dritte, wie Eltern oder Lehrerinnen und Lehrer, kann abhängig von deren Erwartung und Erfahrung variieren. Die Wirkung eines Arzneimittels mag in verschiedenen Anforderungssituationen unterschiedlich erkennbar sein (z. B. Wirkung von Psychostimulanzien). Wie oben beschrieben, sind viele klinische Veränderungen des Kindes und Jugendlichen reifungsabhängig. Das heißt, es kann sich über die Behandlungsdauer eine entwicklungsbedingte Verbesserung oder aber auch eine Verschlechterung der Symptomatik ergeben, die primär nichts mit der Wirkung einer Medikation zu tun haben. In dieser Situation kann es zu falschen Attributionen kommen, so dass ein Medikament infolge seiner zeitlichen Nähe zur Symptomveränderung fälschlicherweise als wirksam, unwirksam bzw. symptomaggravierend eingeschätzt wird.

Viele Kinder und Jugendliche, die medikamentöser Interventionen bedürfen, haben komorbide Störungen: So bestehen oftmals Angststörungen und Depressionen gleichzeitig oder auch ADHS, Störungen des Sozialverhaltens und Depressionen. Hier ist es dann umso wichtiger, Zielsymptome für die Pharmakotherapie eindeutig zu definieren und auch zu operationalisieren, um im Verlauf die Wirkung beurteilen zu können.

Ein zusätzlich erschwerender Faktor für eine rationale Therapie mit Neuro‑/Psychopharmaka bei Kindern und Jugendlichen mit Intelligenzminderung oder Autismus ist das *Underreporting*, welches darin besteht, dass diese Patientengruppe häufig Sprachbarrieren aufweist und damit Wirkung sowie UAWs nur erschwert oder indirekt erkennbar werden. Der oder die Behandelnde muss sich dann auf die Auskunft durch Dritte (Angehörige, Erzieherinnen/Erzieher, Lehrerinnen/Lehrer) verlassen.

### Erstattungs- und haftungsrechtliche Fragen bei der Off-Label-Anwendung

In Deutschland ist die Verordnungsfähigkeit von Arzneimitteln durch die gesetzliche Krankenversicherung (GKV) grundsätzlich von einer Zulassung abhängig. Aufgrund des Wirtschaftlichkeitsgebotes des Sozialgesetzes ist die Krankenkasse nicht zur Kostenübernahme bei der Off-Label-Anwendung eines Arzneimittels verpflichtet. Das bedeutet, Betroffene und Sorgeberechtigte müssen in den Aufklärungsgesprächen darauf hingewiesen werden, dass es erstattungsrechtliche Probleme bei gesetzlich Versicherten geben kann [3, 4, 10]. Es ist oft ratsam, vor jedem Off-Label-Gebrauch einen Antrag bei der jeweiligen Krankenkasse zu stellen und die Behandlung genau zu begründen, jedoch sind die Hürden für eine Erstattung erheblich. Entsprechend eines Urteils des Bundessozialgerichtes vom 19.03.2002 (Az.: B1 KR 37/00 R) ist die Verschreibung von Medikamenten außerhalb ihrer zugelassenen Indikation unter folgender Voraussetzung zulässig und eine Leistung der GKV:

- Vorliegen einer schwerwiegenden, lebensbedrohlichen oder die Lebensqualität auf Dauer nachhaltig beeinträchtigenden Erkrankung, für die keine andere Therapie verfügbar ist,
- die begründete Aussicht aufgrund der Datenlage, dass mit dem Arzneimittel ein kurativer oder palliativer Behandlungserfolg erzielt werden kann, beispielsweise durch Ergebnisse einer Phase-III-Studie oder anderweitig erlangter Erkenntnisse von gleicher Qualität, die einen relevanten Nutzen oder eine relevante Wirksamkeit mit einem vertretbaren Risiko belegen.

Insbesondere in Hinblick auf den letzten Punkt ist es hilfreich, wenn sich Behandelnde auf Leitlinien oder Empfehlungen von Fachgesellschaften oder auf Ergebnisse von Konsensuskonferenzen berufen können.

Neben diesen erstattungsrechtlichen Problemen bei der Off-Label-Anwendung von Neuro‑/Psychopharmaka gibt es Unsicherheiten bezüglich des Haftungsrechts, wobei nach juristischen Einschätzungen die Hersteller für alle Schäden, die bei bestimmungsgemäßem Gebrauch eines Arzneimittels entstehen, haften [12]. Der Begriff „bestimmungsgemäß“ umfasst dabei sowohl Indikationsangaben im Beipackzettel bzw. in Fachinformationen als auch wissenschaftlich anerkannte Therapiegewohnheiten, die nicht als Kontraindikationen ausgeschlossen werden.

Ein möglicher, wenn auch aufwendiger Ausweg aus der rechtlichen Grauzone wäre die Aufnahme von in der Kinder- und Jugendpsychiatrie häufig off-label verwendeten Neuro‑/Psychopharmaka in die Anlage VI der Arzneimittel-Richtlinie [13]. Über die Aufnahme entscheidet der Gemeinsame Bundesausschuss (G-BA) aufgrund der Bewertungen zum Stand der wissenschaftlichen Erkenntnis von den beim Bundesinstitut für Arzneimittel und Medizinprodukte (BfArM) eingerichteten Expertengruppen. Alle diejenigen Arzneimittel, die in Teil A der Anlage VI aufgenommen werden, sind dann verordnungsfähig, wenn das pharmazeutische Unternehmen, das die Zulassung für das entsprechende Arzneimittel besitzt, dem Off-Label-Einsatz zugestimmt und eine entsprechende Haftungsübernahme nach § 84 Arzneimittelgesetz (AMG) abgegeben hat [13].

### Problematik der Durchführung klinischer Studien im Kindes- und Jugendalter

Es gibt vielfältige Gründe, warum nur wenige Neuro‑/Psychopharmaka für die Behandlung von Kindern und Jugendlichen zugelassen sind. Zum einen sind Zulassungsverfahren sehr aufwendig und kostenintensiv. Sie werden deshalb durch die pharmazeutische Industrie vor allem für solche Arzneimittel durchgeführt, die bei häufig vorkommenden Erkrankungen angewendet werden können und somit eine hohe Verordnung zu erwarten ist. Der Absatzmarkt für Neuro‑/Psychopharmaka bei Kindern und Jugendlichen ist aber verhältnismäßig klein. Weitere Hindernisse sind rechtliche, ethische und methodische Probleme bei der Durchführung randomisierter, placebokontrollierter Studien, die ein zentrales Element des Zulassungsverfahren sind, sowie eine fehlende Infrastruktur für die multizentrische Arzneimittelprüfung mit Schwierigkeiten bei der Patientenrekrutierung [14].

Bei klinischen Prüfungen nach dem AMG können Minderjährige sich nicht eigenständig für eine Teilnahme entscheiden, es müssen immer die Sorgeberechtigten zustimmen [3]. Minderjährige in klinischen Studien haben ein Recht auf altersentsprechende Aufklärung, weshalb in der Praxis für sie meist eigene Aufklärungsformulare verwendet werden.

Methodische Probleme bei der Evaluation von klinischen Studien im Kindes- und Jugendalter tragen ebenfalls dazu bei, dass kaum Zulassungsverfahren für Neuro‑/Psychopharmaka angestrebt werden. So gibt es wie oben beschrieben erhebliche Schwierigkeiten, die gewünschte Wirkung eines Arzneimittels nachzuweisen, da Symptome kontext- und reifungsabhängig sein und stark schwanken können. Auch die Beurteilung von Symptomen durch Dritte, wie Eltern oder Lehrerinnen und Lehrer, kann abhängig von deren Erwartung und Erfahrung variieren. Schließlich gibt es Hinweise darauf, dass die Placebo-Responseraten höher sind als bei Erwachsenen. Das bedeutet, dass in pädiatrischen Studien wesentlich mehr Versuchspersonen in die Studien eingeschlossen werden müssen als bei Studien an Erwachsenen [15, 16].

Die schwierige Vorhersage der Dosis-Wirkungs-Beziehung allein auf der Basis von Untersuchungen an Erwachsenen, mögliche unvorhersehbare Arzneimittelwirkungen und Bedenken hinsichtlich möglicher Spätfolgen (z. B. auf Wachstum oder Gehirnentwicklung) führen zu einer Zurückhaltung bei der Durchführung von und Teilnahme an klinischen Studien mit Neuro‑/Psychopharmaka bei Kindern und Jugendlichen. Die limitierten finanziellen Anreize für die Industrie sowie die äußerst begrenzten Möglichkeiten, industriefreie Drittmittel für Zulassungsstudien einzuwerben, führen zu einer höchst unbefriedigenden Verfestigung der gegenwärtigen Off-Label-Praxis. Ärztinnen und Ärzte, die in Kooperation mit der pharmazeutischen Industrie klinische Studien durchführen, sehen sich nicht selten dem Vorwurf ausgesetzt, dass dadurch ihre Therapie- und Verordnungsentscheidungen beeinflusst würden [17].

Obwohl eine Reihe von regulatorischen und finanziellen Maßnahmen in der Europäischen Union (EU) und Nordamerika ergriffen wurde (u. a. EU-Direktive 2001/20/EG von 2001 und EU-Verordnung von 2006, welche 2007 in Kraft trat; Food and Drug Administration Modernization Act von 1997), um die medikamentöse Versorgung und Sicherheit von Kindern und Jugendlichen zu verbessern [15, 16], hat sich wenig hinsichtlich der für Minderjährige zugelassenen Neuro‑/Psychopharmaka geändert. Im Zeitraum 2004 bis 2006 und 2012 bis 2014 gab es in der EU jeweils nur 4 Arzneimittel aus dem ZNS-Bereich, die für Minderjährige zugelassen wurden [18]; zum jetzigen Zeitpunkt ist die Anzahl der im Kindes- und Jugendalter zugelassenen Neuro‑/Psychopharmaka weiterhin sehr übersichtlich [19, 20].

Die ergriffenen Maßnahmen verpflichteten pharmazeutische Unternehmen gesetzlich dazu, klinische Studien im Kinder- und Jugendbereich durchzuführen, wenn ein Arzneimittel für Erwachsene zugelassen werden soll, und einen entsprechenden Prüfplan bei Einreichung des Arzneimittelantrages auf Genehmigung bei der Zulassungsbehörde vorzulegen. Zusätzlich wurden Anreize geschaffen, um die pharmazeutische Industrie zur Durchführung klinischer Studien an Kindern und Jugendlichen anzuregen. Hierzu gehören eine Patentverlängerung um 6 Monate, wenn mit einem bereits zugelassenen Arzneimittel klinische Studien an pädiatrischen Patientinnen und Patienten durchgeführt werden, und eine neue Form der Arzneimittelzulassung, die sogenannte Genehmigung für die pädiatrische Verwendung („paediatric use marketing authorisation“; PUMA). Diese besondere zusätzliche Genehmigung kann für jedes Arzneimittel erteilt werden, welches bereits für Erwachsene zugelassen ist und für das eine weitere Zulassung ausschließlich für die Verwendung in der pädiatrischen Bevölkerung beantragt wird. Obwohl seit der Einführung dieser Maßnahmen der Anteil der an Kindern und Jugendlichen durchgeführten randomisierten, doppelblinden klinischen Studien zwischen 2007 und 2013 auf etwa 10 % anstieg [21], kam es nur zu einer einzigen Zulassung im Rahmen der PUMA [14]. Dies lässt darauf schließen, dass diese regulatorischen Maßnahmen keinen ausreichenden Anreiz für die pharmazeutische Industrie darstellen, um Arzneimittel nach ausgelaufenem Patentschutz für die pädiatrische Anwendung zu entwickeln.

## Fazit

Die medikamentöse Behandlung von psychischen Erkrankungen im Kindes- und Jugendalter stellt vor dem Hintergrund der häufigen Off-Label-Anwendung, entwicklungsabhängiger Modifikationen und aufgrund fehlender Erkenntnisse in Bezug auf die Langzeitwirkungen vieler Neuro‑/Psychopharmaka eine besondere Herausforderung dar. Trotz der Bemühungen in der EU, die Situation durch eine Reihe von regulatorischen und finanziellen Maßnahmen zu verbessern, hat sich hinsichtlich der für Minderjährige zugelassenen Neuro‑/Psychopharmaka wenig geändert.

Um die wissenschaftliche Evidenz für die klinische Anwendung und die langfristige Sicherheit von Neuro‑/Psychopharmaka in der kinder- und jugendpsychiatrischen Praxis zu erweitern, bedarf es weiterer öffentlicher Fördermaßnahmen. Ein Beispiel hierfür ist die durch das BfArM geförderte TDM-VIGIL-Studie, in der Informationen zum Verordnungsverhalten und zur Sicherheit von Psychopharmaka im Kindes- und Jugendalter u. a. durch die Einrichtung eines digitalen Patientenregisters systematisch erfasst werden [22, 23]. Außerdem lässt das AMG auch Studien im Off-Label-Bereich zu, die nicht die Wirksamkeit, sondern Sicherheit untersuchen. Entsprechende Studien benötigen eine eigene öffentliche Förderstruktur, um pharmaunabhängige Evaluationen von Wirkstoffen zu erlauben, für die der Patentschutz ausgelaufen ist und für die kein gesteigertes wirtschaftliches Interesse besteht. Auf Basis dieser Studien sollten begrenzte Zulassungen bei entsprechender klinischer Notwendigkeit ermöglicht werden (vgl. Abschlussbericht KIJU-Aktion Psychisch Kranke; https://www.apk-ev.de/fileadmin/downloads/Materialien_KiJu/Abschlussbericht_APK-Projekt_KiJu-WE_.pdf).

Da randomisierte, kontrollierte Zulassungsstudien in der Regel mit einer relativ kleinen Anzahl, im jeweiligen Studienrahmen selektierter Patientinnen und Patienten sowie einer begrenzten Beobachtungsdauer durchgeführt wurden, müsste ein entsprechendes Register in Deutschland etabliert werden, um belastbarere Daten zur langfristigen Wirkung und Arzneimittelsicherheit zu erhalten.
